# Soil Allies: Exploring the Combined Potential of *Folsomia candida* and *Trichoderma* spp. Against *Fusarium oxysporum*

**DOI:** 10.1111/1758-2229.70202

**Published:** 2025-09-29

**Authors:** Juan Carlos Santos‐Barbosa, Jorge Molina, María X. Rodríguez‐Bocanegra

**Affiliations:** ^1^ Department of Biological Sciences, Centro de Investigaciones en Microbiología y Parasitología Tropical (CIMPAT) Universidad de los Andes Bogota Colombia; ^2^ Department of Microbiology Unidad de Investigaciones Agropecuarias (UNIDIA), Pontificia Universidad Javeriana Bogotá Colombia

**Keywords:** fungi, *Fusarium*, mycophagy, phytopathogen, springtails, synergy, *Trichoderma*

## Abstract

*Fusarium oxysporum* is a widespread phytopathogenic fungus that affects a variety of crops worldwide. This study evaluated the independent effects of three species of *Trichoderma* (*T. harzianum*, *T. viride* , and *T. longibrachiatum*) and the springtail *Folsomia candida* on the suppression of *F. oxysporum* under laboratory conditions. We conducted separate in vitro assays to assess fungal antagonism and feeding preferences of the springtail. The results demonstrated that all *Trichoderma* species significantly inhibited *F. oxysporum* growth, whilst *F. candida* showed a marked preference for consuming *F. oxysporum* mycelium over that of the tested *Trichoderma* species. These findings suggest that both organisms may contribute to the reduction of *F. oxysporum* independently. This preliminary work lays the foundation for future studies investigating potential interactions and combined biocontrol applications under more complex and ecological settings.

## Graphical abstract

This study highlights the synergistic potential of the springtail *Folsomia candida* and *Trichoderma* spp. in suppressing *Fusarium oxysporum*, a key pathogen affecting cape gooseberry. By reducing *Fusarium* populations and enhancing soil health, this approach offers a promising eco‐friendly strategy for managing plant diseases in agriculture.

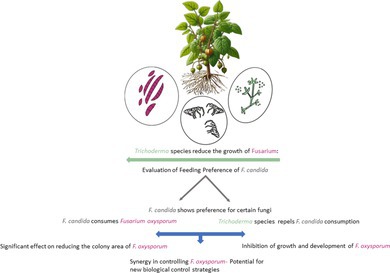

## 1 Introduction

The rhizosphere, as defined by Otten (2008), is a dynamic soil zone influenced by root activity, where bacteria, protists, fungi, and soil invertebrates interact intensively with each other and with the plant. The interactions amongst these organisms transform the structure of the soil and modify biogeochemical cycles affecting the community itself (Siddiky, Kohler, et al. 2012; Larsen et al. 2015). Some of these interactions include symbiosis (mycorrhizae and nitrogen‐fixing bacteria), predation (mites and spiders), herbivory (rhizophagy by coleopteran larvae), parasitism (fungi and bacteria), and mycophagy (nematodes and springtails) (Bonkowski et al. 2009; Johnson and Rasmann 2015).

Amongst the animals that inhabit the rhizosphere, springtails (Order Collembola) are arthropods soil mesofauna considered mainly detritivorous, mycophagous, and occasionally rhizophagous (Johnson and Rasmann 2015). Their roles as selective mycophagous arthropods have been described in detail by several authors (Klironomos et al. 1999; Sabatini and Innocenti 2000a; Larsen et al. 2008; Tordoff et al. 2008). In addition, springtails are one of the most frequent arthropods in edaphic biota (Twardowski et al. 2016). Several important effects on the rhizosphere are associated with springtails, as they introduce soil modifications through aggregates and pores, using exudates such as glomalin, and working in conjunction with other organisms such as mycorrhizae (Klironomos et al. 1999; Siddiky, Schaller, et al. 2012). Furthermore, there is a wide diversity of fungal consumption preferences amongst springtails, which has been documented in several studies. Under laboratory conditions, some species feed primarily on phytopathogens—such as *Rhizoctonia cerealis*, *R. solani* , *Zymoseptoria tritici, Fusarium graminearum*, *F. culmorum*, *Bipolaris sorokiniana*, and *Gaeumannomyces graminis*—rather than on saprophytic or beneficial fungi (Sabatini and Innocenti 2000b; Bourgeois et al. 2023; Shiraishi et al. 2003). Although this predilection may change depending on the interactions between the different species of springtails and fungi (Maraun et al. 2003).

Springtail feeding preferences may be influenced by a wide range of factors. These include the type of substrate (Leonard and Bradbury 1984; Klironomos et al. 1999), vertical distribution within the soil profile (Jørgensen et al. 2003), presence of toxic or deterrent compounds (Shaw 1988; Sadaka‐Laulan et al. 1998; Rohlfs et al. 2007), pigmentation (Thiele and Larink 1990), fungal colony age (Visser and Whittaker 1977), hyphal nutrient content (Booth and Anderson 1979; Verhoef et al. 1988; Scheu and Simmerling 2004), effects on fecundity (Friberg et al. 2005), and the size and morphology of spores (Kaneko et al. 1995). Large populations of springtails can also modulate fungal populations in soils (Hannula et al. 2023). For example, collembola grazing can modify the architecture of the roots, as they prefer to feed on phytopathogenic fungi instead of beneficial fungi, such as mycorrhizae (Bonkowski et al. 2009).

This mycophagy becomes essential when the fungi in the ecosystem act as decomposers, phytopathogens, or symbionts in the rhizosphere (Klironomos et al. 1999; Sabatini et al. 2002; Siddiky, Schaller, et al. 2012). In agricultural soils, where combinations of different fungi are present and springtails have food preferences towards specific soil‐borne phytopathogenic fungi, this interaction may influence the development of the plant (Sabatini and Innocenti 2000a; Tordoff et al. 2008). In some cases, springtails reduce the disease severity by feeding on fungi (Sabatini et al. 2002). For instance, experiments with the springtail *Folsomia candida* have shown a preference for phytopathogenic fungi like *Alternaria alternata* over beneficial fungi such as mycorrhizae or *Trichoderma* (Klironomos et al. 1999). This preference in *F. candida* may be influenced by olfactory signals (Staaden et al. 2011) or deterrent factors such as toxins, hyphae thickness, or the production of repellent substances (Böllmann et al. 2010). *Folsomia candida* is widely used as a model organism due to its common presence in soil ecosystems, ease of maintenance in laboratory settings, and parthenogenetic reproduction, which enables rapid population growth and consistent genetic backgrounds. Moreover, the interactions of *F. candida* with soil fungi provide valuable insights into soil ecology and biological control mechanisms (Fountain and Hopkin 2004).

On the other hand, *Fusarium oxysporum* is a soil‐borne pathogen with wide ecological plasticity and a wide spectrum of plant hosts of agricultural importance (Altinok and Erdoğan 2015; Kala et al. 2016; Toghueo et al. 2016). *F. oxysporum* affects cape gooseberry crops ( *Physalis peruviana* ) in Colombia, causing damping‐off and vascular wilt with millionaire losses during exportation (Osorio‐Guarín et al. 2016). Colombia is the largest cape gooseberry producer in the world, and cape gooseberry is the fifth most exported fruit (Minagricultura 2022).

The disease caused by *F. oxysporum* starts with the presence of hyphae, conidia, or chlamydospores in the soil or harvest residues that germinate when they are activated by the exudates produced by fibrous roots (Kraft and Pfleger 2001; Akhter et al. 2016). The germinative tubes of the fungus penetrate the root epidermis by wounds and then colonise the vessels of the xylem, producing occlusion of the vascular system of the plant (Kraft and Pfleger 2001). As a result, a brown coloration is produced in both roots and stems, followed by chlorosis, loss of turgor (leaf shrinkage), poor fruit development, and finally, plant death (Zapata et al. 2002; Swarupa et al. 2014). The infection rate is determined by factors such as the time of the initial infection, virulence, and climatic conditions; depending on the infection rate, the fungus can cause radicular rot and death, even in young plants (Agrios 2005). Current methods to deal with *Fusarium* include the application of chemical fungicides such as triazoles, with combinations like Propiconazole and Prochloraz showing high efficacy in inhibiting fungal growth (Mondani et al. 2021).

In this context, new phytosanitary management focuses on environmentally friendly practises using agents as biocontrollers with low effects on soil biodiversity (Toghueo et al. 2016; Jaiswal et al. 2022). Several rhizospheric organisms have been used against *F. oxysporum* as biocontrollers, including fungal species such as *Trichoderma* (Urrea et al. 2011; Altinok and Erdoğan 2015; Kala et al. 2016; Toghueo et al. 2016). *Trichoderma* is a cosmopolitan and opportunistic ascomycete that uses antagonism and competition to modulate interactions with other microorganisms in the rhizosphere (Woo et al. 2023). The colonisation of plants on the roots as an endophyte also produces multifaceted benefits for plants and makes *Trichoderma* a microorganism contributing to eco‐sustainable agriculture (Woo et al. 2023).

Because root diseases caused by fungi are highly difficult to control (Altinok and Erdoğan 2015), and knowing their impact on Colombian agriculture (Zapata et al. 2002; Urrea et al. 2011); in this study, we evaluated the effect of *Folsomia candida* as a selective mycophagous agent on *Fusarium oxysporum*, using a strain isolated from an infected *Physalis peruviana* crop. Given the known antagonistic activity of *Trichoderma* spp. against soil‐borne pathogens, we hypothesise that the combined presence of both organisms may reduce the presence of the soil‐borne pathogen *F. oxysporum*. This potential interaction warrants further investigation under more complex experimental conditions.

## 2 Materials and Methods

### 2.1 Springtails and Fungi

The collembola were kindly donated by the Behavioural Ecophysiology and Herpetology Group (GECOH) at Universidad de los Andes, Bogotá. The species was determined to be *Folsomia candida* Willem 1902 according to the keys of Ospina et al. (2009) and Janssens (1996). *F. candida* is a well‐known cosmopolitan species (Fountain and Hopkin 2004). The springtails were reared before testing at 20°C in plastic jars containing a sterilised substrate based on charcoal and coconut husk, and moistened with water weekly. The animals were also fed weekly with a mixture of yeast, rice, chickpea, bread, pasta, and fish food, powdered and mixed in equal proportions (modified from Seres et al. 2018). Only adults were used (over 21 days of age).

*Fusarium oxysporum* isolated from a *P. peruviana* crop and three species of *Trichoderma* (*T. harzianum*, *T. viride* , and *T. longibrachiatum*) were obtained from the fungal collections of the Agricultural Research Unit (UNIDIA) at Pontificia Universidad Javeriana, and the Laboratory of Mycology and Phytopathology (LAMFU) at Universidad de los Andes, respectively. The fungi were activated in Petri dishes containing potato dextrose agar (PDA) and maintained at 28°C ± 2°C.

### 2.2 Antagonistic Effect of *Trichoderma* spp. on *F. oxysporum*

The antagonistic effects of the three *Trichoderma* species against *F. oxysporum* were evaluated in vitro using the dual culture technique (Coşkuntuna and Özer 2008). *Trichoderma* species and *F. oxysporum* were grown separately in PDA for seven days at 28°C ± 2°C. The inoculum of each fungus was prepared as a concentrated conidial suspension in sterilised distilled H_2_O (10^6^ conidia/ml). A 10 μL aliquot of each *Trichoderma* isolate inoculum was placed on the surface of PDA plates at a 1.0 cm distance from the edge of the Petri dish, and a 10 μL aliquot of *F. oxysporum* inoculum was placed on the opposite side of the Petri dish also at a 1.0 cm distance from the edge of the plate. Four repetitions per treatment were performed with the combination biocontrol agent (*Trichoderma* spp.) and pathogenic fungus (*F. oxysporum*) by following the methodology proposed by Coşkuntuna and Özer 2008. Controls included *F. oxysporum* inoculated alone on the surface of PDA medium at a 1.0 cm from the edge of the Petri dishes, as well as each *Trichoderma* species inoculated alone under the same conditions. These controls allowed us to compare radial growth patterns and assess antagonistic interactions in co‐culture assays. Both, the dual and control cultures, were incubated at 28°C ± 2°C and checked daily during four days for growth speed and conidiation. The antagonistic effect of *Trichoderma* spp. was determined on the fourth day as the decrease in mycelial growth of *Fusarium oxysporum* using the following formula:

$$\text{Antagonistic effect}=\left(A-\frac{B}{A}\right)x\;100$$

Abd‐El‐Khair et al. (2010) where *A* is the growth area of *F. oxysporum* control (mm^2^). *B* is the growth area of *F. oxysporum* mycelium facing *Trichoderma* spp. (mm^2^).

The growth areas of every fungus treatment were measured from pictures taken daily until the fourth day by using the software Image J (Schneider et al. 2012). The growth rate was calculated using the radial growth rate formula (Trinci 1971):

Growth rate (*K*) was defined as

$$K=\frac{\left(R_{1}-R_{0}\right)}{\left(t_{1}-t_{0}\right)}$$

where *R*_1_ is the last measure of fungal growth area. *R*_0_ is the first measure of fungal growth area. *t*_1_ is the last day of measure. *t*_0_ is the first day of measure.

### 2.3 Feeding Preference Test by *Folsomia candida*

The methodology to evaluate the fungal biomass feeding preference by *F. candida* springtails was followed according to the approach proposed by Sabatini and Innocenti (2000b), with some modifications: Petri dishes of 90 mm diameter with 20 mL of water agar (WA) were inoculated with a 1.0 cm diameter PDA‐mycelial plugs of both fungal species. The two fungal inocula were placed each 2.0 cm from the edge of the Petri dish on opposite sides (each *Trichoderma* spp. isolate faced with *F. oxysporum*). PDA‐mycelium plugs from two incubation periods (4 and 10 days) were used. PDA plugs without fungi were used as controls when exposed to either *F. oxysporum* or *Trichoderma* spp. Eight replicas at four different time periods per treatment were performed (biocontrol agent *Trichoderma* spp. vs. pathogenic fungus *F. oxysporum*), as well as for each control.

Fifteen adult animals kept without food for 48 h before the beginning of the test were introduced using a fine brush in the centre of the Petri dishes at the same distance from the two choices. All test plates were maintained at 20°C, and to evaluate the possible thigmotropic effect on springtails, holes without agar were made facing plugs of WA. The preference of consumption was determined based on the specific location of each one of the individuals at different times: 1, 2, 24, and 48 h after starting the experiment.

### 2.4 Fungal Area Consumption Test by *Folsomia candida*

To determine the consumption of fungal biomass area in colonies of *F. oxysporum* and each of the three *Trichoderma* spp. isolates, 15 collembolan adults were allowed to feed for 35 days on cultures of 8 days for each fungus growing in Petri dishes with PDA with 14 replicates for each fungus. To determine the total fungal biomass consumed in each colony by the collembolans, we measured the differences in areas of fungal biomass from pictures taken before and after 5 weeks by using ImageJ (Schneider et al. 2012).

After each treatment of fungal area consumption and feeding preference test, pools of springtails from each experiment were collected in jars for evaluation by clearing the cuticle or by PCR amplification.

#### 2.4.1 Cuticle Clearing

After 48 h of fungal consumption in the feeding preference tests and fungal area consumption ended, all individuals of *F. candida* were removed from the Petri dishes and their cuticle was cleared following the methodology recommended by Young and Duncan (1994) with KOH 10% for 18 h; and then, preserved in phenol. The cleared cuticle allowed us to observe the interior of the collembolan intestine under an optical microscope, with the fungal biomass consumed.

#### 2.4.2 Molecular Confirmation

Fungal cultures of *Fusarium oxysporum* and each of the three *Trichoderma* spp. isolates were incubated in PDA at 28°C ± 2°C. From each fungus, mycelia were grown in liquid GYEP medium, and their DNA was extracted using the Zymo Quick‐DNA Faecal/Soil Microbe Kit. For collembolan DNA, the Zymo Quick‐DNA Tissue/Insect Kit was used following the recommended protocol. In all cases, DNA concentration was quantified by using a nanodrop and compared with a size/mass molecular weight marker before PCR amplification. DNA extraction was performed immediately after visual evaluation was completed (cuticle clearing). The Zymo Quick‐DNA Faecal/Soil Microbe Kit was also used to extract fungal DNA from the guts of *F. candida* .

For PCR amplification, the primers ITS4 and ITS5, described by White et al. (1990), were used to amplify a fragment of rDNA. PCR amplifications were performed in a total volume of 25 μL by using the Promega GoTaq Green Master Mix by mixing 40 ng of the template DNA with 0.5 mM of each primer. These reactions were carried out in a Bio‐Rad T‐100 thermal cycler with an initial denaturation of 5 min at 95°C, followed by 35 cycles of 1.5 min at 94°C, 2 min at 55°C, and 3 min at 72°C, with a final extension of 5 min at 72°C. DNA fragments (7 μL) were analysed by electrophoresis on 1.5% (w/v) agarose gel in 1× TAE buffer (stained with Sybersafe from Invitrogen) and photographed (BioRad gel documentation system). The molecular size marker was Hipperlader II (50 bp) from Bioline.

### 2.5 Statistical Analysis

For the fungal antagonism and consumption assays, analyses were conducted using PRISM 9.4.1 for Windows, with data normality assessed through the Shapiro–Wilk test. In the fungal antagonism test, ANOVA was used to detect significant differences in growth area means, followed by Tukey's test for post hoc comparisons. For the fungal area consumption test, differences in fungal areas were identified using the Kruskal–Wallis test. In the preference test, the statistical methodology of Bourgeois et al. (2023) was applied, with analyses performed in R (version 4.1.1; R Core Team 2021). To assess fungal plug selection, a logistic regression model with a binomial distribution (presence ~ Time + Days + FungalSpecies −1) was employed, excluding the intercept to enable predictions solely based on the specified predictors and provide clearer insights into their effects. The fungal species on the plug (*Trichoderma* spp. or *F. oxysporum*) was treated as a categorical fixed effect. Generalised linear mixed effect models (GLMM), based on Bolker et al. (2009) and using the lme4 package (Bates et al. 2007), were employed with replicates included as a random effect. Proportion data were analysed assuming a binomial distribution with a logit link function. All generalised linear models were validated using the DHARMa package (Hartig 2017), assessing scaled residuals, distribution fit, overdispersion, and outliers.

## 3 Results

### 3.1 Antagonistic Effect of *Trichoderma* spp. on *F. Oxysporum*

**FIGURE 1:**
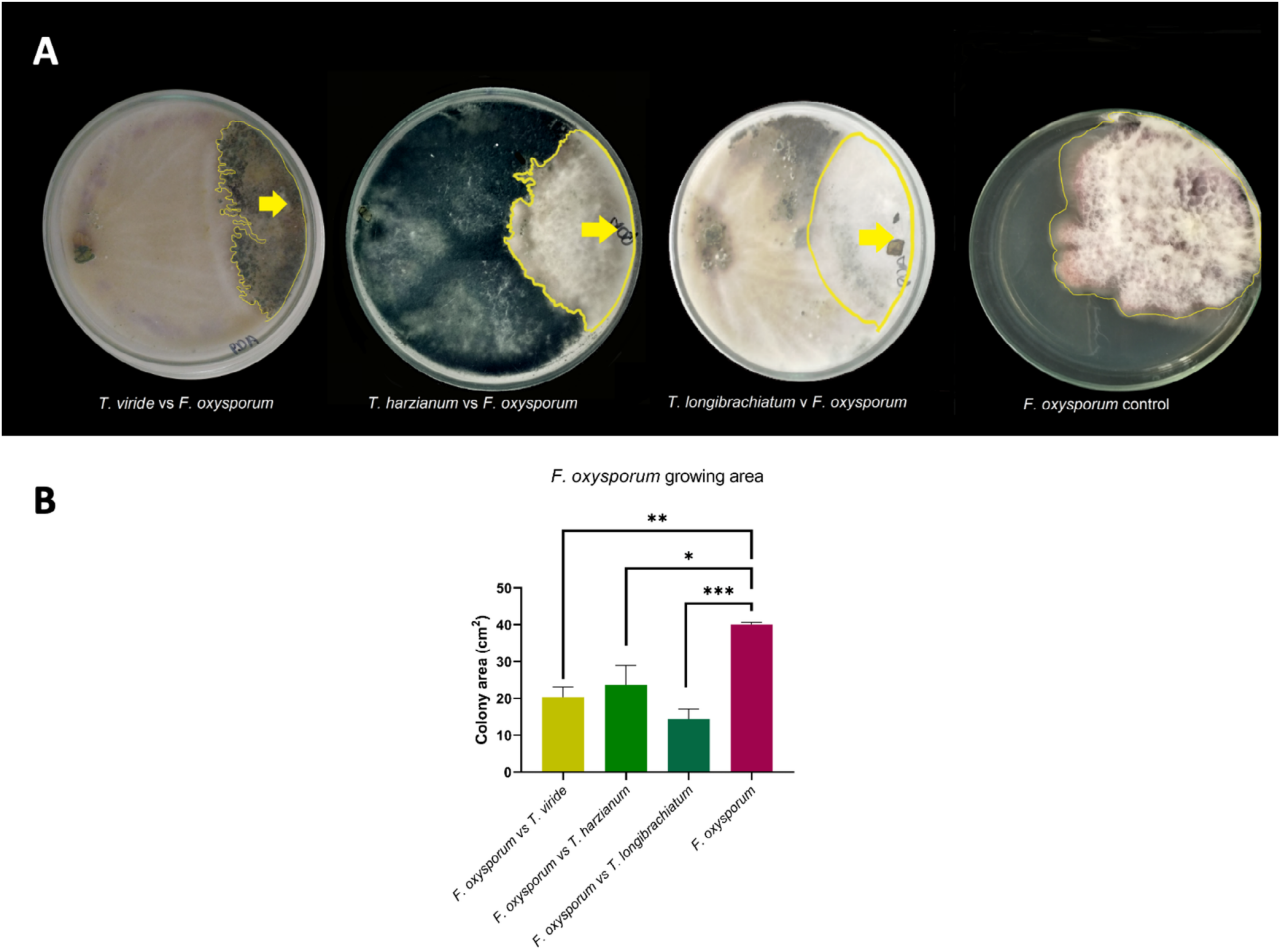
Antagonistic effect of *Trichoderma* spp. on *Fusarium oxysporum*. (A) Pictures showing the growth inhibition in *F. oxysporum* (highlighted with a yellow line) faced with *Trichoderma* spp. Some *Trichoderma* spp. can be observed inside the mycelia of *F. oxysporum* (see arrows). (B) Reduction in colony area of *F. oxysporum* due to the effect of the *Trichoderma* species. Asterisk are showing significant differences amongst treatments.

The results of dual culture indicated that *Trichoderma* spp. isolates ( *T. viride* , *T. harzianum*, *T. longibrachiatum*) inhibited the growth of *F. oxysporum* by 40.12% ± 0.1%, 38.69% ± 0.1%, and 35.02% ± 0.1% (mean ± SD), respectively (Figure 1A). The average growth area of *F. oxysporum* in dual cultures with *T. viride* , *T. harzianum*, and *T. longibrachiatum* was 6.8 ± 0.9 cm^2^, 7.0 ± 1.4 cm^2^, and 7.4 ± 1 cm^2^ (mean ± SD), respectively (Figure 1B). A significant difference was found between the control and the three *Trichoderma* spp. treatments (*p* < 0.05, ANOVA test). In addition, the growth rate of all *Trichoderma* spp. isolates was higher in comparison with *F. oxysporum* (14.4, 17.0, and 14.7 cm^2^/day for *T. viride* , *T. harzianum*, and *T. longibrachiatum*, respectively; versus 2.4 cm^2^/day of *F. oxysporum*). The growth rate amongst *Trichoderma* species was not significantly different (*p* > 0.05, ANOVA test). In contrast, the growth rate of *F. oxysporum* against the three *Trichoderma* species was significantly different from that of the control (*p* < 0.05, ANOVA test).

### 3.2 Feeding Preferences in *Folsomia candida*

**FIGURE 2:**
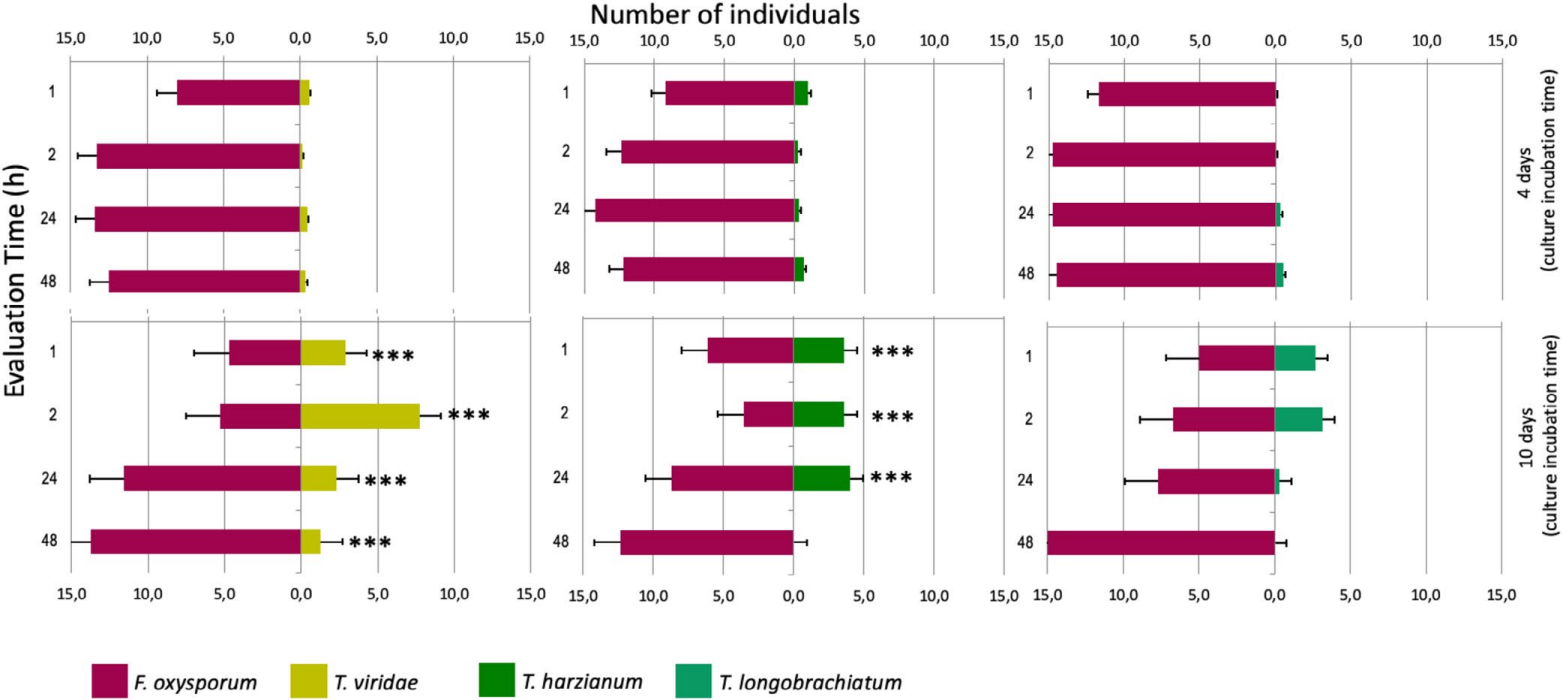
Preferences in fungal consumption by *Folsomia candida* in dual choice experiments facing *Fusarium oxysporum* against different species of *Trichoderma* with 4 and 10 days of incubation time.

In total, 3240 *F. candida* individuals were evaluated in the preference tests, and 1080 springtails were evaluated for each preference test. At the end, 2795 (86%) springtails showed any preference; that is, they chose one of the two fungi offered after 120 min (2 h) time. In all treatments, *F. candida* showed a preference for the mycelia of *F. oxysporum* (Figure 2). Time (h) was not a statistically significant variable (*p* > 0.05) in any test. However, the time variable (colonies that grew for 4 or 10 days) was statistically significant for *T. harzianum* and *T. viride* (*p* < 0.05), but not for *T. longibrachiatum*. The impact of *T. harzianum* and *T. viride* on the probability of preference was highly significant in the model (*p* < 0.05), with a negative coefficient. In contrast, *T. longibrachiatum* had a very high negative coefficient, but this was not significant. The coefficient for *F. oxysporum* is positive, suggesting that this fungus has a higher probability of preference than any of the *Trichoderma* species tested. However, this effect was not statistically significant (*p* > 0.05), although 2722 (84%) springtails selected this fungus as a food source.

Our analysis also revealed that the attractiveness of *F. oxysporum* to *F. candida* varied with the age of the fungal colony. Younger colonies (4 days old) elicited different responses compared to older ones (10 days), particularly in comparisons involving *T. harzianum* and *T. viride* . This observation suggests that fungal ageing may modulate volatile profiles or surface properties relevant to collembolan foraging behaviour. Additionally, the colony age influenced the feeding preferences, with younger fungal colonies eliciting stronger attraction responses from *F. candida* compared to older colonies, suggesting variation in volatile compound production over time.

### 3.3 Fungal Area Consumption Test

**FIGURE 3:**
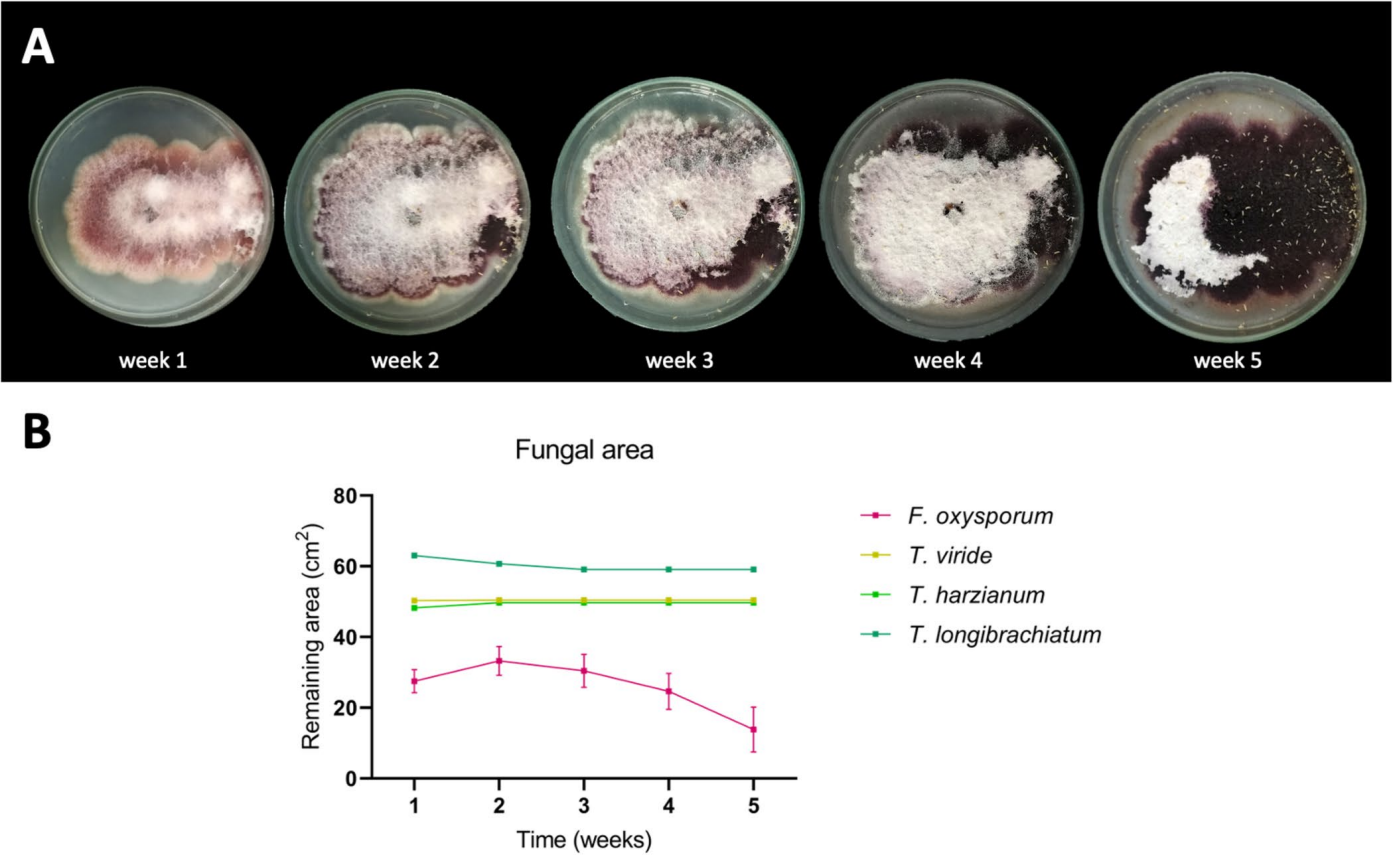
Fungal area consumption by *Folsomia Candida* . (A) Pictures showing fungal area consumption of *Fusarium oxysporum* by the springtail *F. candida*. From left to right pictures are showing graphically the consumption of the same mycelia along five consecutive weeks. (B) Average remaining mycelial area during five weeks of every fungi consumed by *F. candida* .

The consumption of *F. oxysporum* mycelium by *F. candida* was evident, as the area with mycelia decreased over the course of the experiment (Figure 3A). However, the mycelia of Trichoderma spp. remained unconsumed (Figure 3B).

### 3.4 Confirmation of Fungal Biomass Consumption

**FIGURE 4:**
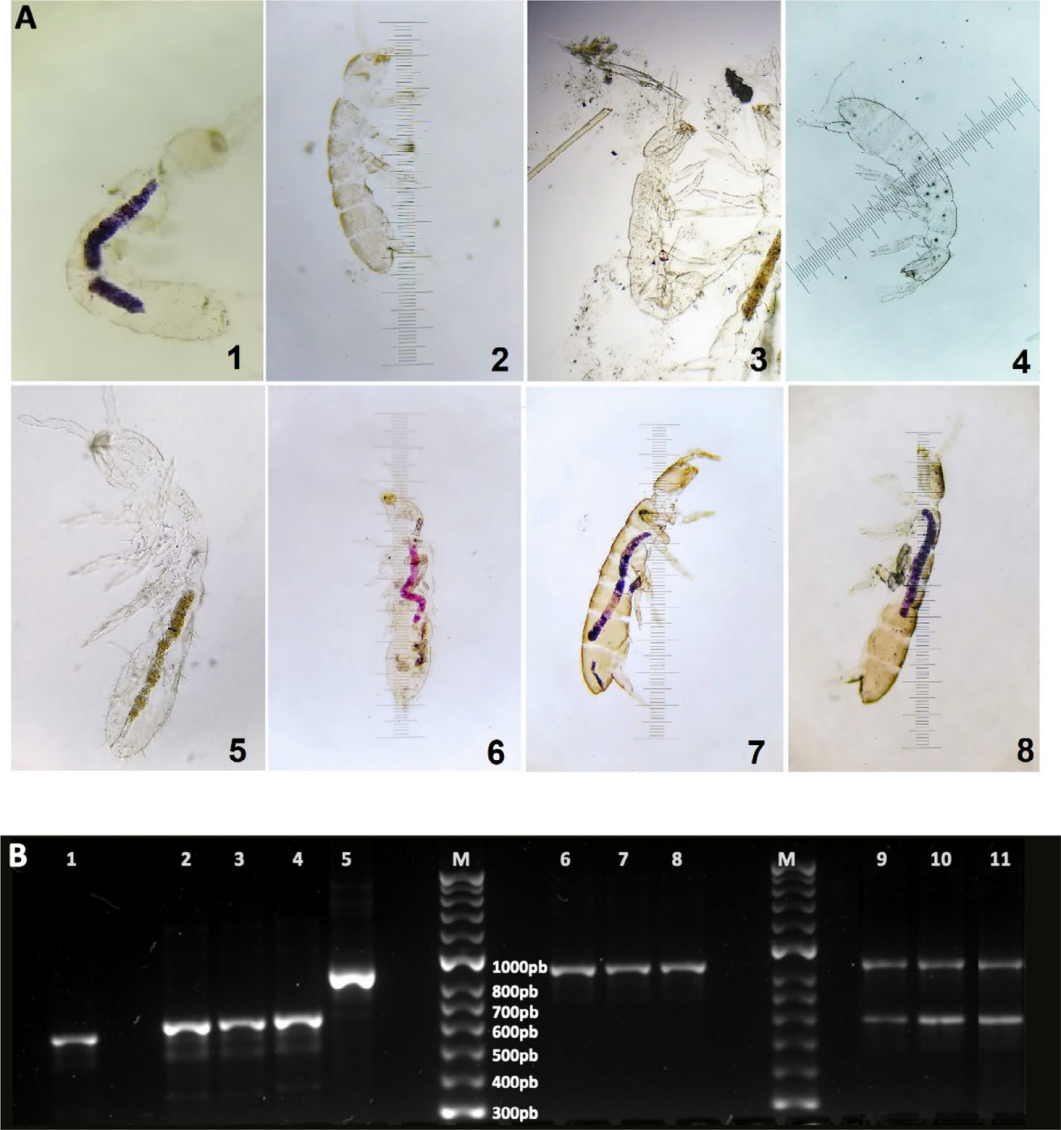
(A) Optical microscopy data through the cleared cuticle of springtails. **1**. *F. candida* exposed to a *F. oxysporum* culture. **2**. *F. candida* exposed to a *T. viride* culture. **3**. *F. candida* exposed to a *T. harzianum* culture **4**. *F. candida* exposed to a *T. longibrachiatum* culture **5**. *F. candida* fed with a yeast mixture. **6**. *F. candida* choosing between *F. oxysporum* and *T. viride* , **7**. *F. candida* choosing between *F. oxysporum* and *T. harzianum* and **8**. *F. candida* choosing between *F. oxysporum* and *T. longibrachiatum*. (B) Polymerase chain reaction (PCR) amplification. **1**. *F. oxysporum* DNA positive control, **2**. *T. viride* DNA positive control **3**. *T. harzianum* positive control. **4**. *T. longibrachiatum* DNA positive control, and **5**. *F. candida* DNA positive control. **6–8**. DNA Extracted from *F. candida* exposed to the different *Trichoderma* cultures. In all these cases, no DNA from *Trichoderma* sp. was amplified (**6**. *T. viride* , **7**. *T. harzianum*, and **8**. *T. longibrachiatum*) (no DNA from *Trichoderma* sp. was amplified). **9–11**. DNA Extracted from *F. candida* in the preferences experiments. **9**. *F. oxysporum* /*T. harzianum*, **10**. *F. oxysporum* / *T. viride* , and **11**. *F. oxysporum*/*T. longibrachiatum*. Again, in all cases, no DNA from *Trichoderma* sp. was amplified. M. Molecular weight markers DNA.

Purple colour characteristics of *F. oxysporum* colonies were observed in the intestines of several *F. candida* individuals exposed to colonies of *F. oxysporum* (Figure 4A). *F. candida* fed with yeast and other ingredients showed a brown colour in the gut (Figure 4A, picture 5), and springtails allowed to feed on colonies of *Trichoderma* spp. had no evidence of fungal biomass within the stomodeum (Figure 4A, pictures 2–4). A reddish‐purple coloration can be seen inside the animals exposed to the preference experiment (*F. oxysporum*—*Trichoderma* spp.) (Figure 4A, pictures 6–8) due to feeding on *F. oxysporum*, as observed when the springtails were exposed only to *F. oxysporum* culture (Figure 4A, picture 1).

PCR results confirmed the consumption of fungal biomass by *F. candida* in *F. oxysporum* (Figure 4B). The fragments amplified using the ITS primers for *Trichoderma* spp. had an approximate size of 563–602 bp (Figure 4B, lines 2, 3, and 4) (Hermosa et al. 2002; Devi et al. 2012), whereas *Fusarium* has an approximate size of 550 bp (Figure 4B, line 1) (Chehri et al. 2011). The DNA amplification product of *F. candida* has an approximate size of 900 bp (Figure 4B, line 5). DNA extracted from the feeding preference experiments showed that in treatments combining *F. oxysporum* with any *Trichoderma* species, the DNA amplification product of *Trichoderma* was not observed (Figure 4B, lines 9, 10, 11). In these cases, when *F. candida* was exposed to any *Trichoderma* species growing alone, only DNAs from the springtails were amplified (Figure 4B, lines 6, 7, 8). These molecular data corroborate the selective feeding behaviour observed, confirming the ingestion of *F. oxysporum* biomass and the avoidance of *Trichoderma* spp. by *F. candida* .

These observations support the notion that behavioural avoidance of *Trichoderma* by *F. candida* is mediated by chemical signals, and that selective feeding patterns may have ecological implications for fungal community dynamics in soil.

## 4 Discussion

### 4.1 Feeding Preferences in *Folsomia candida*

Our in vitro experimental approaches, including observation of intestinal contents, molecular confirmation, and behavioural assays, confirmed that *F. candida* displays a clear feeding preference for the phytopathogenic fungus *F. oxysporum* over the three tested *Trichoderma* species ( *T. viride* , *T. longibrachiatum*, and *T. harzianum*).

Although collembolans such as *F. candida* avoid *Trichoderma* spp., likely because of their high chitinolytic activity or toxic metabolites (Lartey et al. 1989; Bourgeois et al. 2023), they often prefer phytopathogens such as *Fusarium* spp. and *Rhizoctonia solani* in food‐choice experiments (Xu et al. 2019). This differential grazing behaviour may reduce foraging pressure on beneficial antagonists, allowing them to persist longer in the rhizosphere and maintain their biocontrol activity by influencing fungal community composition in the soil. Their feeding activity can either promote dominant or weakly competitive fungi or, at higher densities, suppress overall fungal biomass (Crowther et al. 2012; Hannula et al. 2023). The ability of collembolans to modulate the proliferation of phytopathogenic fungi has been demonstrated in vitro, including the reduction in the biomass of *Zymoseptoria tritici* and *F. graminearum* under moderate grazing conditions (Bourgeois et al. 2023). The effect of collembolan density is key; whilst low to moderate densities may stimulate fungal growth, high densities often result in biomass suppression (Crowther et al. 2012; Hannula et al. 2023).

Goncharov et al. (2020) recognised that *Fusarium* species are preferable as a nutritious food source by promoting the growth of most studied species, especially for the springtail *F. candida* . As collembola feed directly on fungal mycelia, food choice may be influenced by different mechanisms, such as nutrient richness, low C:N ratio, lipid acids, or hyphal morphology, as in the case of *Fusarium* species (Goncharov et al. 2020). *Fusarium* has been previously shown to be used as food by other springtails, such as *Protaphorura armata* (Sabatini et al. 2004). However, in our experiments, *F. candida* fed on fungi depending on the age of the *F. oxysporum* colony (Figure 2). A similar effect was reported by Sabatini and Innocenti (2000b) for the interactions between the fungus *Bipolaris sorokiniana* and springtail *Onychiurus pseudogranulosus*. Bengtsson et al. (1991) observed that VOCs changed with the age of the culture, as well as the growth substrates used for the fungal species *Mortierella isabellina* and *Verticillium bulbillosum*.

The attraction of *F. candida* to *F. oxysporum* is likely mediated by volatile organic compounds (VOCs), which act as infochemicals and chemical signals that guide invertebrate foraging decisions (Sawahata et al. 2008; Becher et al. 2018; Holighaus and Rohlfs 2019). Collembola, including *F. candida* and *Onychiurus armatus* , can detect and discriminate fungal odours, particularly eight‐carbon volatile oxylipins commonly produced by fungi (Visser and Whittaker 1977; Bengtsson et al. 1991). These VOCs vary with fungal species and are influenced by the physiological state and substrate composition. In addition, fungal age plays a crucial role in VOC production: younger, actively growing colonies tend to emit more attractive compounds, whereas older colonies show reduced emissions and less attractive profiles (Holighaus and Rohlfs 2016; Rohlfs et al. 2007).

Fungi often synthesise repellent or toxic secondary metabolites as a defence mechanism against fungivory. For example, *F. candida* exhibits strong avoidance behaviour towards strains of *Aspergillus nidulans* that produce elevated levels of secondary metabolites, whilst preferring laeA‐knockout strains with reduced metabolite production (Döll et al. 2013). Likewise, grazing by collembolans has been shown to induce the synthesis of pigments such as aurofusarin that in *Fusarium* species deter further feeding without being overtly toxic (Xu et al. 2019). Melanin synthesis genes may also influence food quality and defence because pigmented fungi tend to be more chemically defended (Rohlfs et al. 2007).

Volatile organic compounds (VOCs) such as sesquiterpenes and ketones produced by *Trichoderma* spp. function as repellents to fungivores (Poveda 2021; Ghosh et al. 2021). Supporting this, Slonka et al. (2024) showed that *T. virens* mutants deficient in sesquiterpene synthesis exhibited increased attractiveness to *F. candida* , underscoring sesquiterpenes' role in mediating fungivore avoidance. Despite avoidance behaviour, no significant differences were found in collembolan fitness parameters, suggesting that these VOCs act primarily as behavioural repellents rather than toxins. Overall, these findings support the hypothesis that *Trichoderma's* influence on fungivore behaviour is chemically mediated and may contribute to its success as a biocontrol agent by reducing grazing pressure from fungivores such as *F. candida* .

As a fungivorous arthropod, *F. candida* likely relies on volatile organic compounds (VOCs) emitted by fungi as chemical signals to assess their toxicity and feeding suitability. In the case of *Trichoderma* spp., these VOCs may function as repellent infochemicals that elicit avoidance behaviour in collembolans (Holighaus and Rohlfs 2016; Sawahata et al. 2008; Slonka et al. 2024; Staaden et al. 2011). This repellent function is distinct from deterrents that inhibit feeding after contact. Instead, VOCs act as early‐stage cues, prompting fungivores to avoid potentially harmful fungi (Holighaus and Rohlfs 2019; Bengtsson et al. 1991; Hedlund et al. 1995).

This chemical signaling system allows springtails to avoid the ingestion of harmful fungal metabolites. These cues may direct them away from valuable fungal structures, such as fruiting bodies, cleistothecia, or sclerotia, which are often rich in protective secondary metabolites (Döll et al. 2013; Sawahata et al. 2008; Holighaus and Rohlfs 2019), enhancing their survival and steering foraging behaviour. Some of these toxins, such as aurofusarin and other polyketides, are known to affect a wide range of organisms. Fungal species that are typically rejected by collembolans often contain compounds that are toxic to humans and other animals (Friberg et al. 2005; Holighaus and Rohlfs 2016).

This pattern supports the notion that fungal toxins possess broad‐spectrum defensive properties that protect fungi from diverse predators and competitors in the soil environment (Trienens and Rohlfs 2011; Ruiz‐Jiménez et al. 2019; Urbaneja‐Bernat et al. 2020; Xu et al. 2019). The consistent avoidance of *Trichoderma* spp. by *F. candida* , despite the absence of lethal or sublethal effects in some cases (Slonka et al. 2024), suggests that springtails are behaviourally tuned to chemical cues rather than relying solely on post‐ingestive feedback.

*F. candida* , likely detects fungal VOCs through its olfactory system using specialised receptor neurons located in its antennae (Becher et al. 2018; Hedlund et al. 1995; Holighaus and Rohlfs 2019; Slonka et al. 2024). Electroantennogram recordings have demonstrated that different fungal VOCs elicit differential responses in collembolans, such as *Orchesella cincta* and *Tomocerus flavescens* , depending on the fungal species involved, including *Cladosporium herbarum* , *Mortierella isabellina*, and *Penicillium spinulosum* (Hedlund et al. 1995). However, VOCs are not the only factors that shape the behaviour of fungivores.

Species of *Trichoderma* are consistently avoided by collembolans and often lead to reduced survival and reproduction when they are the sole food sources (Sadaka‐Laulan et al. 1998; Lartey et al. 1989; Slonka et al. 2024; Bourgeois et al. 2023). This pattern is attributed to the presence of a broad range of toxic or dissuasive metabolites concentrated in propagules and mycelia (Altinok and Erdoğan 2015; Poveda 2021; Sood et al. 2020). These include hydrolytic enzymes such as chitinases, which may damage the arthropod cuticle (Bourgeois et al. 2023), as well as antifungal peptaibols and VOCs with repellency or anti‐feeding effects (Monte 2023; Slonka et al. 2024). *Trichoderma* appears to involve primarily the action of bioactive metabolites rather than pigmentation alone (Scheu and Simmerling 2004; Slonka et al. 2024).

Taken together, the combination of chemical defences, including enzymes, volatile repellents, and non‐volatile toxicants, likely explains the complete lack of foraging on *Trichoderma* spp. observed in our study (Figure 2) (Staaden et al. 2011; Rohlfs et al. 2007; Döll et al. 2013). These interactions highlight the evolutionary and functional consequences of chemical defence in shaping fungivore feeding behaviour.

Our results showing no feeding by *F. candida* on any of the *Trichoderma* species tested here (Figure 3B) are consistent with previous reports. Hannula et al. (2023) noted that *Trichoderma* spp. can be lethal or lead to starvation in collembolans owing to the high chitinolytic activity in their hyphae, whilst Lartey et al. (1989) observed high mortality in springtails fed with *T. harzianum*. Similarly, Bourgeois et al. (2023) reported avoidance behaviour of *Heteromurus nitidus* towards chitinase‐producing fungi, including *Trichoderma*. This genus is also known to produce entomopathogenic metabolites, antifeedant compounds, and repellents (Ghosh et al. 2021; Poveda 2021; Monte 2023), which may contribute to its unpalatability.

Further studies are required to determine whether the feeding behaviour of *F. candida* observed here (Figures 2, 3, 4) is influenced by VOCs or non‐volatile organic compounds (nVOCs) associated with the physical characteristics of fungi (such as pigments and hyphae thickness).

### 4.2 Antagonistic Effect of *Trichoderma* spp. on *F. oxysporum*

*Trichoderma* is well known for its antagonistic properties, as observed here in the reduced growth area of *F. oxysporum* (Figure 1). This genus is widely employed as a biocontrol agent owing to its multiple antagonistic mechanisms, including mycoparasitism, production of antibiotics and other antifungal compounds, secretion of chitinases, glucanases, and proteases, competition for space and nutrients, and antibiosis (Dennis and Webster 1971; Kubicek et al. 2001; Verma et al. 2007; Vinale et al. 2007; Saba 2012; Adnan et al. 2019; Sood et al. 2020; Tyskiewicz et al. 2022). Previous studies have shown an antagonistic effect of *Trichoderma* spp. on *F. oxysporum*, but the degree of reduction in pathogen growth may vary (El‐Katatny et al. 2007; Coşkuntuna and Özer 2008; Perveen and Bokhari 2012; Sundaramoorthy and Balabaskar 2013; Altinok and Erdoğan 2015). Also, previous studies have demonstrated the good performance of *T. harzianum*, *T. viride* , and *T. longibrachiatum* as antagonists of *F. oxysporum* (Abdelrahman et al. 2016; Javanshir Javid et al. 2016; Kala et al. 2016).

*Trichoderma* can also outcompete other microorganisms, including plant pathogens, for nutrients and space in the soil, which is attributed to its rapid growth rate and efficient nutrient uptake (Tyskiewicz et al. 2022). The ability of *Trichoderma* to utilise a wide range of carbon and nitrogen sources gives it an advantage over other microorganisms with more specific nutrient requirements (Woo et al. 2023).

In our experiments, the growth rates of the three *Trichoderma* species were much faster than that of *F. oxysporum*, suggesting that competition for nutrients and space was the main mechanism of antagonism. However, other antagonistic mechanisms, such as mycoparasitism and volatile and non‐volatile metabolites, may also operate simultaneously. As reviewed by Tyskiewicz et al. (2022), *Trichoderma* species exhibit a diverse arsenal of antagonistic strategies, including mycoparasitism, secretion of hydrolytic enzymes (chitinases, glucanases, and proteases), and production of antifungal metabolites. The presence of *Trichoderma* hyphae within *F. oxysporum* colonies (Figure 1A) may indicate mycoparasitic activity. Then, the reduction in the growth area of *F. oxysporum* by *Trichoderma* (Figure 1) is likely due to a combination of these factors. Further research is required to determine the specific mechanisms underlying these fungal interactions.

Interestingly, the three *Trichoderma* species evaluated (*T. harzianum*, *T. viride* , and *T. longibrachiatum*) showed a comparable antagonistic effect on *Fusarium oxysporum*, reducing its growth in similar proportions under in vitro conditions. This convergence across distinct species within the same genus suggests that antagonistic traits may be conserved amongst *Trichoderma* spp., at least in their interactions with *F. oxysporum*. Such consistency supports the genus‐level selection of *Trichoderma* as a reliable group of biocontrol agents, offering flexibility in practical applications and reinforcing its potential in integrated pest management strategies.

### 4.3 Complementary Roles of *Trichoderma* spp. and *Folsomia candida*

In addition to their direct antagonistic action against phytopathogens, *Trichoderma* spp. may also influence the behaviour of non‐target soil fauna, including fungivorous arthropods such as collembolans. Wen et al. (2020) showed that *T. longibrachiatum* and *T. harzianum* reduced the repellency of the entomopathogenic fungus *Metarhizium anisopliae* towards subterranean termites, allowing the termites to tolerate or approach zones that they would normally avoid. However, this altered behaviour was not necessarily beneficial to the termites; increased exposure to *M. anisopliae* may elevate the risk of infection, highlighting the complex and potentially maladaptive consequences of behavioural interference.

This type of behavioural modulation, observed in other soil fauna, invites parallels with our findings on collembolans. Considering our findings, the selective feeding behaviour of *F. candida* , which has shown a marked preference for *F. oxysporum* and other phytopathogens whilst generally avoiding *Trichoderma* spp., may indirectly promote the establishment and activity of beneficial fungi in the rhizosphere. This avoidance behaviour is likely driven by the production of chitinolytic enzymes and antifungal secondary metabolites, such as peptaibols, sesquiterpenes, and hydrolytic compounds like chitinases, which are known to repel collembolans or negatively impact their survival and reproduction (Sadaka‐Laulan et al. 1998; Lartey et al. 1989; Poveda 2021; Slonka et al. 2024; Bourgeois et al. 2023). Repellent effects may stem from volatile signals, such as those mediated by sesquiterpenes (Slonka et al. 2024), and ingestion‐based toxicity, particularly in species with high chitinolytic activity.

In contrast, *F. candida* has been shown to reduce the biomass of phytopathogens, such as *Fusarium culmorum*, in infected wheat straw, particularly under moderate grazing intensities that do not eliminate fungal growth entirely (Crowther et al. 2012; Meyer‐Wolfarth et al. 2017; Wolfarth et al. 2013). By selectively grazing on susceptible fungi whilst avoiding antagonistic species such as *Trichoderma*, collembolans may shift competitive balances in the soil microbial community, alleviating pressure on beneficial fungi and enhancing their persistence and colonisation potential in the rhizosphere.

These ecological cascades, driven by selective fungivory, highlight the potential for complementary or even synergistic interactions in multi‐agent biocontrol systems. Although our experimental design did not include formal long‐term tripartite assays, we observed no antagonism between *F. candida* and *Trichoderma* spp. Instead, their mechanisms, antagonistic inhibition by *Trichoderma*, and selective grazing on *F. oxysporum* by *F. candida* appear ecologically compatible. This suggests the possibility of additive or synergistic effects under more complex conditions arising from the simultaneous suppression and consumption of pathogens.

Under greenhouse and field conditions, *Trichoderma* species, including those tested in this study, have consistently demonstrated the ability to control a wide range of plant diseases and enhance crop productivity and quality (Tyskiewicz et al. 2022). Additionally, the combined application of *Trichoderma* and beneficial bacteria has proven effective against *F. oxysporum* in tomato crops (Rini and Sulochana 2007). Therefore, the integration of *Trichoderma* spp. and *F. candida* may offer novel and promising biocontrol strategies that reduce dependence on agrochemicals. To confirm such synergy, future studies should include long‐term tripartite or microcosm designs that reflect realistic soil food webs and microbial dynamics in greenhouse and field settings to unravel the dynamics of *Trichoderma*–collembolan interactions within the rhizosphere communities.

## 5 Conclusions

Biological control strategies that integrate multiple soil organisms are increasingly relevant for sustainable agriculture and the management of phytopathogens. This study demonstrates, through in vitro assays, that both *Folsomia candida* and three species of *Trichoderma* (*T. harzianum*, *T. viride* , and *T. longibrachiatum*) independently suppressed the growth of *Fusarium oxysporum*, a major phytopathogen affecting *Physalis peruviana* . Whilst no formal interaction assays were performed, our observations suggest that the combined presence of these organisms may yield complementary effects through fungal inhibition and selective mycophagy.

These findings underscore the importance of evaluating the ecological roles of individual soil organisms, as well as their potential interactions, before designing integrated biocontrol strategies. Importantly, the behavioural avoidance of *Trichoderma* spp. by *F. candida* , together with its preference for *F. oxysporum*, highlights a potential ecological synergy that could enhance the persistence of beneficial fungi in the rhizosphere.

However, laboratory conditions offer simplified systems that may not fully represent the complexity of soil communities. Further studies are required under greenhouse and field conditions to assess the interactive dynamics of *F. candida* and *Trichoderma* spp., ideally using long‐term, tripartite, or microcosm experiments that reflect realistic biotic and abiotic interactions in the rhizosphere.

## Author Contributions

**Juan Carlos Santos‐Barbosa:** conceptualization, investigation, writing – original draft, methodology, writing – review and editing, software, data curation. **Jorge Molina:** supervision, resources, investigation, conceptualization, funding acquisition, formal analysis, project administration, writing – review and editing. **María X. Rodríguez‐Bocanegra:** conceptualization, investigation, writing – original draft, validation, methodology, writing – review and editing, formal analysis, data curation, supervision.

## Conflicts of Interest

The authors declare no conflicts of interest.

## Data Availability

The data that supports the findings of this study are available in the Supporting Information of this article.
